# The challenge of preventing and containing outbreaks of multidrug-resistant organisms and *Candida auris* during the coronavirus disease 2019 pandemic: report of a carbapenem-resistant *Acinetobacter baumannii* outbreak and a systematic review of the literature

**DOI:** 10.1186/s13756-022-01052-8

**Published:** 2022-01-21

**Authors:** Reto Thoma, Marco Seneghini, Salomé N. Seiffert, Danielle Vuichard Gysin, Giulia Scanferla, Sabine Haller, Domenica Flury, Katia Boggian, Gian-Reto Kleger, Miodrag Filipovic, Oliver Nolte, Matthias Schlegel, Philipp Kohler

**Affiliations:** 1 Division of Infectious Diseases and Hospital Epidemiology, Cantonal Hospital St. Gallen, St. Gallen, Switzerland; 2 Division of Human Microbiology, Center for Laboratory Medicine, St. Gallen, Switzerland; 3 Division of Infectious Diseases and Hospital Epidemiology, Cantonal Hospital Muensterlingen, Muensterlingen, Switzerland; 4 Division of Intensive Care Medicine, Cantonal Hospital St. Gallen, St. Gallen, Switzerland

**Keywords:** COVID-19, Multidrug-resistant organisms, Outbreaks, Review

## Abstract

**Background:**

Despite the adoption of strict infection prevention and control measures, many hospitals have reported outbreaks of multidrug-resistant organisms (MDRO) during the Coronavirus 2019 (COVID-19) pandemic. Following an outbreak of carbapenem-resistant *Acinetobacter baumannii* (CRAB) in our institution, we sought to systematically analyse characteristics of MDRO outbreaks in times of COVID-19, focussing on contributing factors and specific challenges in controlling these outbreaks.

**Methods:**

We describe results of our own CRAB outbreak investigation and performed a systematic literature review for MDRO (including *Candida auris)* outbreaks which occurred during the COVID-19 pandemic (between December 2019 and March 2021). Search terms were related to pathogens/resistance mechanisms AND COVID-19. We summarized outbreak characteristics in a narrative synthesis and contrasted contributing factors with implemented control measures.

**Results:**

The CRAB outbreak occurred in our intensive care units between September and December 2020 and comprised 10 patients (thereof seven with COVID-19) within two distinct genetic clusters (both ST2 carrying OXA-23). Both clusters presumably originated from COVID-19 patients transferred from the Balkans. Including our outbreak, we identified 17 reports, mostly caused by *Candida auris* (n = 6) or CRAB (n = 5), with an overall patient mortality of 35% (68/193). All outbreaks involved intensive care settings. Non-adherence to personal protective equipment (PPE) or hand hygiene (n = 11), PPE shortage (n = 8) and high antibiotic use (n = 8) were most commonly reported as contributing factors, followed by environmental contamination (n = 7), prolonged critical illness (n = 7) and lack of trained HCW (n = 7). Implemented measures mainly focussed on PPE/hand hygiene audits (n = 9), environmental cleaning/disinfection (n = 9) and enhanced patient screening (n = 8). Comparing potentially modifiable risk factors and control measures, we found the largest discrepancies in the areas of PPE shortage (risk factor in 8 studies, addressed in 2 studies) and patient overcrowding (risk factor in 5 studies, addressed in 0 studies).

**Conclusions:**

Reported MDRO outbreaks during the COVID-19 pandemic were most often caused by CRAB (including our outbreak) and *C. auris.* Inadequate PPE/hand hygiene adherence, PPE shortage, and high antibiotic use were the most commonly reported potentially modifiable factors contributing to the outbreaks. These findings should be considered for the prevention of MDRO outbreaks during future COVID-19 waves.

**Supplementary Information:**

The online version contains supplementary material available at 10.1186/s13756-022-01052-8.

## Introduction

Multidrug-resistant organisms (MDRO) pose an ever increasing threat on health-care systems around the world. Inadequate hand hygiene, high-level use of broad-spectrum antibiotics, patient comorbidities, and use of medical devices are known risk factors for colonization or infection with MDRO [1, 2]. The notion of both inciting and moderating effects of the COVID-19 pandemic on the incidence of MDRO has been suggested [3, 4]. Factors which might facilitate the spread of MDRO include altered characteristics of health-care worker (HCW)-patient contact, inadequate adherence to PPE, low staffing, breaches in environmental cleaning and inadequate antibiotic use [5–7]. On the other hand, increased awareness regarding hand hygiene and other infection control measures during the pandemic have been reported, which might result in a reduction in spread of these pathogens, particularly in settings with low incidence of COVID-19 [8].

During the second wave of the pandemic (September-December 2020), we experienced an outbreak of a carbapenem-resistant *Acinetobacter baumannii* (CRAB) on the intensive care units (ICU) of our tertiary care centre. CRAB is a globally emerging healthcare-related pathogen, which is endemic in many countries of Southern Europe [9]. In Switzerland, only sporadic *A. baumannii* infections and outbreaks have been reported, mostly as a result of importation from high-risk countries [10, 11].

Here, sharing our own experience and those of others, we aimed at describing features of MDRO (including *C. auris*) outbreaks during the COVID-19 pandemic, and at characterizing challenges in outbreak prevention and management specific to the exceptional conditions faced during the pandemic.

## Methods

### Study design and ethics

This is an outbreak report and a systematic review of the literature concerning MDRO outbreaks during the COVID-19 pandemic. The outbreak is reported according to the ORION statement [12], the review according to the Preferred Reporting Items for Systematic Reviews and Meta-Analyses (PRISMA) statement, where applicable [13]. The review protocol was registered on Prospero (Registration number: 259474) [14].

### Outbreak investigation

We retrospectively studied a CRAB outbreak that involved the medical and surgical ICU in our 700-bed tertiary hospital in Eastern Switzerland. Both COVID-19 and non-COVID-19 patients were treated on the two affected ICUs. Due to the high number of patients during the pandemic, bed capacities were extended from 12 to 16, and from 24 to 32 beds for the medical and surgical ICU, respectively. Cases were defined as patients with CRAB isolation in any kind of sample, including clinical and screening specimens. Patient characteristics including hospital mortality were collected from chart reviews; the impact of CRAB infection (non-related, partially related, and directly attributable) on in-hospital mortality was assessed.

The outbreak investigation was led by a multidisciplinary infection control team (physicians, nurses, hospital epidemiologists) and consisted of contact screenings (defined as sharing the room with a CRAB patient for ≥ 24 h), weekly cross-sectional screening of all ICU patients, routine screenings at day 5 and 10 after ICU discharge for all patients with a stay longer than 48 h on either ICU, and sampling of medical equipment, patient environment, and sinks. Patient screening sites included rectum, skin (axilla and groin), urine (if catheter in situ), and the respiratory tract (if ventilated or in case of tracheostomy)[15]. The further containment strategy included cohorting of CRAB positive patients, intensification of environmental cleaning, observations and training regarding correct use of PPE and hand hygiene performance, and restriction of carbapenem use. We used descriptive statistics to summarize patient characteristics.

### Microbial culture and gram-negative MDRO detection

Copan eSwabs™ (Copan, Brescia, Italy) were used for swabbing and sent to the laboratory immediately after sampling. Enrichment broth (Trypticase Soy Broth (TSB); Becton Dickinson, Sparks, MD, USA) was inoculated with 10 μl of the liquid medium and incubated overnight. Of the enriched broth 10 ul were inoculated onto a chromID ESBL and chromID OXA-48 (bioMérieux, Marcy l'Etoile, France) with a WASP instrument (Copan, Brescia, Italy). After incubating the plates for 19 h in the smart incubators of a WASPLab™ high-resolution images of media plates were inspected using the WASPLab™ WebApp software. Colonies, indicative for gram-negative MDRO were identified for further processes. Identification was done with a MALDI-ToF instrument using the BDAL 9.0 database (MALDI Biotyper Smart System, Bruker Daltonics, Bremen, Germany) with the colony transfer method (“direct smear”). A BD™ Phoenix instrument (Becton Dickinson, Sparks, MD, USA) with NMIC-417 cartridge was used for susceptibility testing. Antimicrobial susceptibility testing (AST) data were interpreted according to respective EUCAST guidelines (version 10.0 in 2020, AST data not shown). Colonies were further tested with Carba NP (bioMérieux, Marcy l'Etoile, France) and with ESBL CT/CTL, TZ/TZL and PM/PML epsilometer (E-) tests (bioMérieux, Marcy l'Etoile, France) according to accredited procedures.

### Whole genome sequencing

DNA was quantified by using the Qubit dsDNA BR HS Assay Kit and Qubit fluorometers (Invitrogen, https://www.thermofisher.com). WGS was performed using Illumina MiSeq with the Nextera XT library preparation kit (Illumina Inc., USA), according to the manufacturer’s procedure. Trimming and assembly of raw reads was performed using the Velvet assembler of the SeqSphere software (Ridom, https://www.ridom.de, version 8.0.1 using *A.baumanii* cgMLST v1.3). The analysis included MLST and cgMLST (2`390 targets). For detecting beta-lactam resistance genes (e.g. OXA-23) ResFinder on the CGE Website was used (http://www.genomicepidemiology.org/). Coverage was at least 25-fold. We defined cgMLST clusters as groups of isolates with ≤ 10 different SNPs between neighbouring isolates. To generate phylogenetic SNP trees, we used SeqSphere (Ridom; Münster, Germany) in the pairwise ignore missing values mode and an unweighted pair group method. The WGS data has been submitted to NCBI under the following submission number PRJNA778060.

### Systematic review: study criteria

Studies reporting MDRO outbreaks in healthcare settings including multidrug resistant (MDR) gram negative bacteria (defined as *Enterobacterales*, *Acinetobacter baumannii* and *Pseudomonas aeruginosa* with resistance to at least 3 of the following antibiotic groups: antipseudomonal penicillins, third-generation cephalosporins, carbapenems, aminoglycosides and fluorochinolones), extended-spectrum beta-lactamase (ESBL) or carbapenemase producers, vancomycin-resistant *Enterococci* (VRE), methicillin-resistant *Staphylococcus aureus* (MRSA), and *Candida auris* in patients of any age between December 2019 and March 2021 were included. We considered studies from peer-reviewed journals and pre-prints, regardless of interventional or observational design, such as cohort and case–control studies, outbreak reports, case-series, research letters or editorials, and epidemiologic surveys. Conference abstracts and studies strictly reporting laboratory and no clinical data were excluded.

### Search methods

Literature research was conducted by a scientific librarian in PubMed, EMBASE, CINHAL, MEDLINE, Cochrane and the NIH iSearch COVID-19 Portfolio engines with screening of cross-references and was restricted to documents in English, German, Spanish, Italian and French language. Applied search terms were [“COVID-19”, “SARS-CoV-2”, “pandemic”, “Coronavirus”] AND [“Multidrug-resistant organisms”, “ESBL-E”, “CPE”, “Carbapenem-resistance”, “Acinetobacter baumannii”, “Candida auris”, “Vancomycin-resistant Enterococci”, “Methicillin-resistant *Staphylococcus aureus*”, “Pseudomonas aeruginosa”], and the respective acronyms (Additional file 1: Table S1).

### Screening of records

Initial screening by titles and abstracts and identification of studies that met inclusion- and exclusion criteria were done by eight authors (RT, MSe, GS, SH, DF, DV, PK, MSch). Studies meeting the inclusion criteria were subjected to full text review by two independent reviewers (RT and MSe); any disagreement was solved by a third arbiter (PK). Duplicates were excluded through automated deduplication by the librarian, initial screening and full text review.

### Data extraction

Data extraction was again performed by two independent reviewers (RT and MSe), with a third as arbiter (PK). The following data were extracted from studies in the review: name of first author, time span (month/year to month/year) and country of outbreak, setting (acute care, intensive care, long-term care, other), ward type (COVID-19 *vs.* non-COVID-19 ward), causing organism and mechanism of resistance, proof of clonality (resistance pattern, pulsed-field gel electrophoresis (PFGE), whole-genome sequencing (WGS)), number of affected patients (colonized or infected; clinical or screening samples), COVID-19 status of patients and patient outcome (i.e. hospital mortality). Furthermore, we assessed factors which potentially promoted and factors which might have helped in containing the outbreaks, all according to the respective study authors.

### Risk of bias

Quality of reporting in included studies was evaluated independently by six authors (RT, MSe, GS, SH, DF, PK) according to an adapted version of the ORION statement [12]. The ORION statement consists of a 22-item checklist and a summary table. We selected 14 items which could be applied to all studies in our review (Additional file 1: Figure S1). Because no established cut-off exists to rate the quality of the studies based on the adapted ORION statement, risk of bias analysis was purely descriptive.

#### Statistical analysis

Due to the descriptive nature of the study, no quantitative analysis was conducted.

## Results

### Outbreak detection and patient characteristics

The first CRAB was isolated in the medical ICU on September 30th 2020 from the blood of a COVID-19 patient repatriated from Northern Macedonia, 11 days after a negative admission screening. An outbreak investigation was started on October 11th, after a second patient tested positive for CRAB in a bronchial fluid sample taken during routine diagnostic bronchoscopy. Screening of 93 contact patients revealed seven positive cases. Until the end of December 2020, one additional patient was transferred from the Balkans to the surgical ICU with a positive admission screening for CRAB, resulting in a total of 10 cases (thereof 7 COVID-19 patients) (Table 1). Eight patients were hospitalized on the medical, and two on the surgical ICU. CRAB was detected mainly in respiratory specimens (7/10); 5 patients were deemed to be infected with CRAB, whereof 3 died as a direct consequence of infection.

**Table 1:** Characteristics of patients and CRAB-isolates from the two ICUs. Patients No. 2 and 9 represent index patients on medical- and surgical ICU

Patient no	Age, gender	ICU	Comorbidities	Admission diagnosis	Location of 1st CRAB detection	ICU-LOS	Outcome
1	66, M	Medical	Acute renal failure, AF, anemia	Severe pancreatitis	BAF	53	Deceased
2	65, M	Medical	BSI, chronic renal failure, CAD, DM, Hepatopathy	COVID-19	Blood cultures	18	Deceased
3	62, F	Medical	Acute renal failure, ARDS, malnutrition, anemia, NASH, DM	Boodstream infection	Rectal swab	43	Discharged alive
4	54, M	Medical	DM, anemia, RDS	COVID-19	BAF	32	Discharged alive
5	60, M	Medical	ARDS, angioedema	COVID-19	Resp. secretions	14	Discharged alive
6	73, M	Medical	ARDS, DM, CAD	COVID-19	BAF	31	Deceased
7	65, M	Medical	Chronic renal failure, HIT, cardiopathy, DM, Malnutrition	Ischemic strokes	BAF	5	Deceased
8	55, M	Medical	ARDS, colitis, DM	COVID-19	Resp. secretions	30	Discharged alive
9	54, M	Surgical	ARDS, ACS, Hyperthyroidism	COVID-19	Axillary swab	7	Deceased
10	68, M	Surgical	BSI, ARDS, ACS, AF, bronchial carcinoma, DM,	COVID-19	Resp. secretions	10	Deceased

*AF* Atrial flutter, *BAF* Broncho-alveolar fluid; *BSI* Blood stream infection, *CAD* Coronary atery disease, *DM* Diabetes mellitus, *ARDS* Acute respiratory distress syndrome, *NASH* Non-alcoholic fatty liver disease, *HIT* Heparine-induced thrombocytopenia, *ACS* Acute coronary syndrome

### Microbiological analyses

**Fig. 1:**
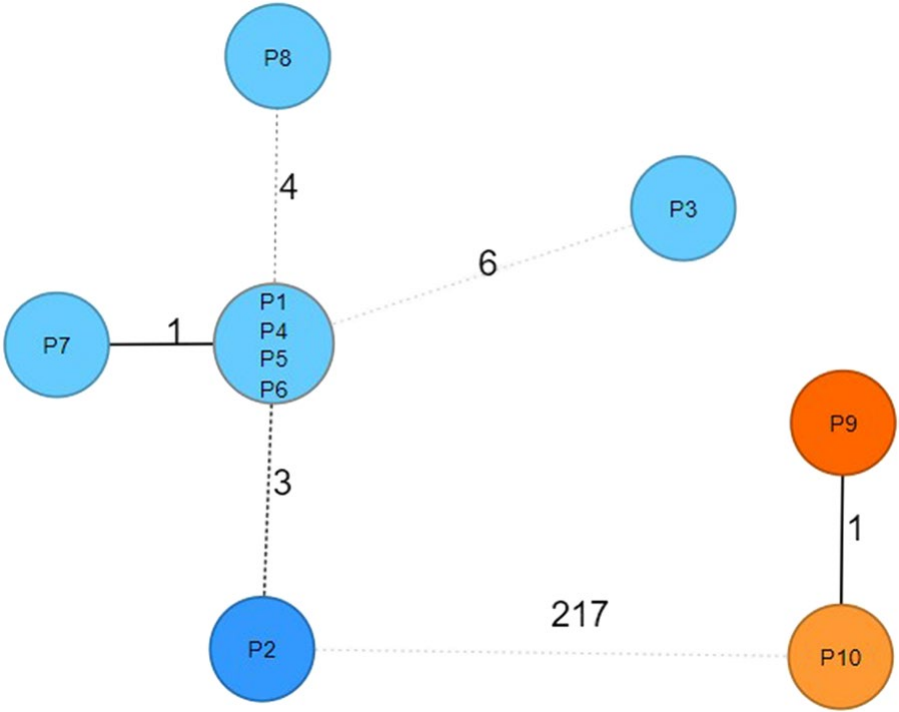
Phylogenetic SNP tree of the outbreak clusters, generated by SeqSphere using the pairwise ignore missing values mode and an unweighted pair group method. P2 and P9 represent two patients transferred from the Balkans (index patients of the two clusters). Blue = Cluster 1 (medical ICU); Orange = Cluster 2 (surgical ICU)

Antimicrobial resistance testing showed that all 10 isolates were resistant to carbapenems (MICs imipenem and meropenem > 8 mg/l). Using single nucleotide polymorphism (SNP) analysis two distinct clusters could be identified (Fig. 1). The larger outbreak showed 4 isolates with identical cgMLSF pattern (i.e. 0 SNPs). On a longitudinal axis, the number of SNPs increased over a period of 65 days, with the highest SNP-count of 9 (compared to the presumed index) in the last isolate of that cluster. CRAB isolates could be attributed to two separate outbreaks, a larger cluster with eight patients and another cluster with two patients. All isolates were of cgMLST sequence type 2, with OXA-23 as carbapenem-resistance conferring enzyme. In both clusters, the first isolate was detected in patients transferred from the Balkans.

### Further outbreak investigations

**Fig. 2:**
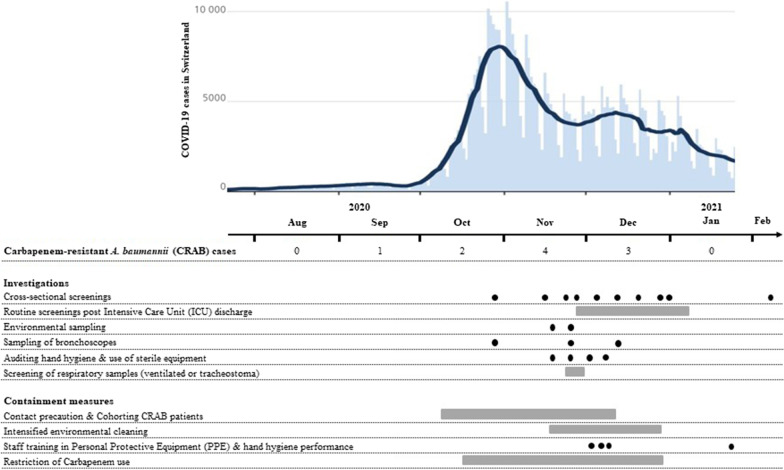
Timeline showing COVID-19 cases in Switzerland, number of CRAB cases per month and chronology of investigation- and containment measures

Sampling of bronchoscopes and of all other environmental samples (bronchoscope storage tubes, glove dispensers, ultrasound, computer keyboards, laryngoscope, sinks; n = 15) remained negative. HCW observations revealed inadequate handling of PPE, especially non-sterile gloves (e.g. no hand hygiene before gloving, gloving without indication, double gloving and disinfection of gloves).We performed four staff training sessions regarding correct use of PPE and hand hygiene on both ICUs. Environmental cleaning was intensified. Restriction of carbapenem use on the medical ICU led to a 91% reduction between September (28.3 DDD/bed) and December 2020 (2.5 DDD/bed). No further cases were detected after December 28th 2020 (Fig. 2). Outbreak investigations were stopped after three negative cross-sectional screenings did not reveal any more cases.

### Studies included in systematic review

**Fig. 3:**
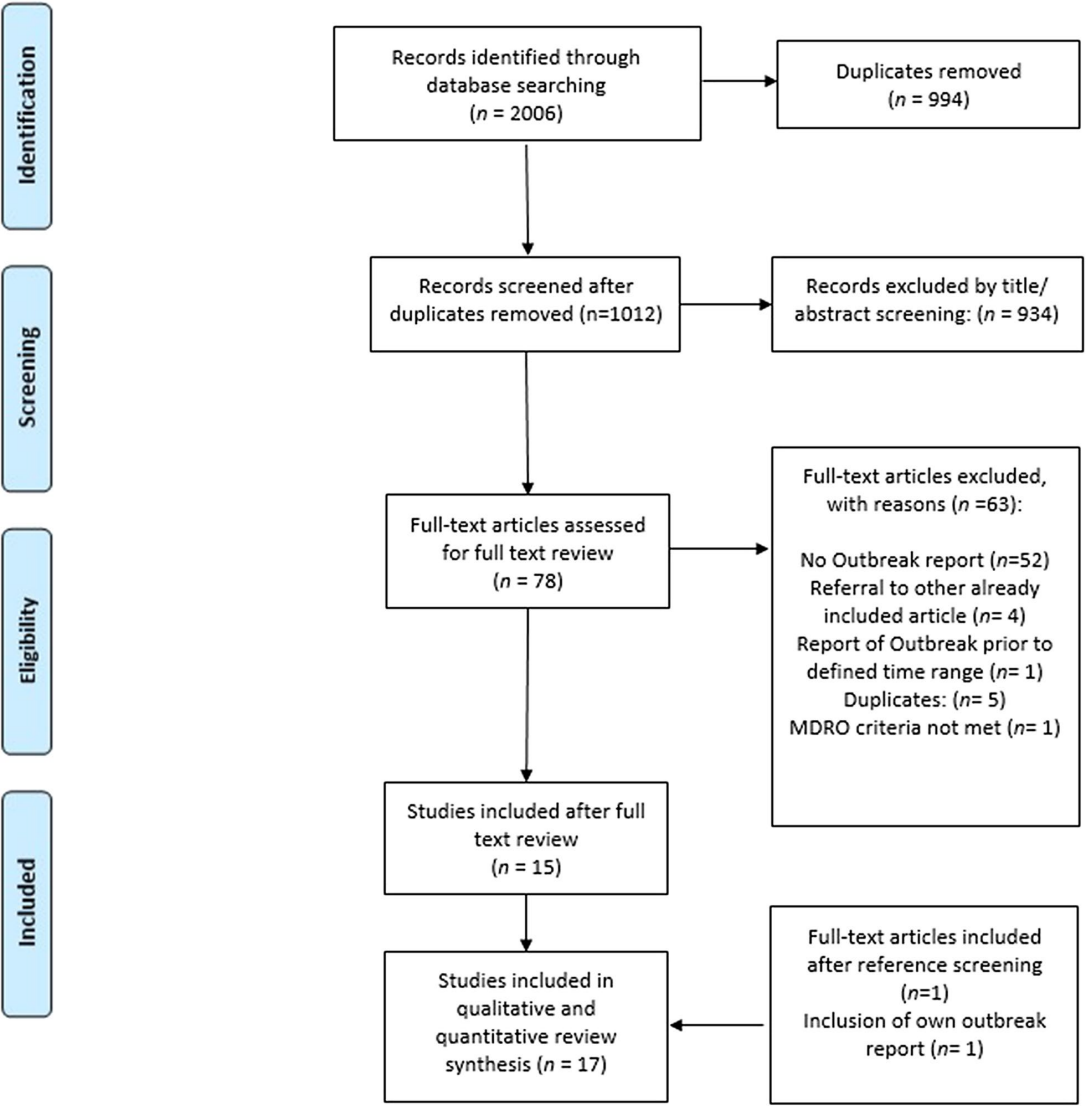
PRISMA flow diagram of the study selection

We identified 2006 articles during the search process. After removal of duplicates 1012 studies remained; 934 articles were excluded during title and abstract screening and 78 studies underwent full-text review. Thereof, 15 studies fulfilled inclusion criteria (the initial inter-reviewer disagreement on 2 studies could be solved upon intervention of the third arbiter). Our own CRAB report as well as one additional record identified through reference screening [16] were also included, resulting in 17 records with a total sample size of 218 patients (Fig. 3).

Six of the 17 included studies were conducted in Europe, four in the United States and four in South-America. Outbreaks were caused by *C. auris* (n = 6) [17–22], *A. baumannii* (n = 5) [5, 14, 16, 23, 24], MDR *Enterobacterales* (n = 5) [25–29]*,* and VRE (1) [30]*.* All outbreaks involved intensive care settings; 186/218 (85%) patients were positive for SARS-CoV-2. A median of 10 patients (range 4–35) were affected per outbreak. In-hospital mortality (reported by 15 studies) was 35% (68/193) (Table 2).

**Table 2:** Summary of the outbreak characteristics in the included studies

Article	Type of article	Time span of outbreak	SARS-CoV-2	Setting	Type of MDR Strain	Size	Patient outcome (hosp. mortality)
Farfour et al. (2020)—France	Original article	03/20–04/20	Positive	ICU	*E. Coli* NDM-5, CTX-M-15	6	0/6
Artega-Livias et. Al. (2021) – Peru	Letter to the Editor	08/20–09/20	Positive	ICU	*K. pneumoniae* NDM, CTX-M	4	3/4
Kampmeier et al. (2020) – Germany	Original ariticle	03/20–04/20	3/5 positive	ICU	*E. faecium* VRE Van B	5	Unknown
Gottesman et al. (2021) Israel	Original article	03/20–04/20	Positive	ICU/Ward	CRAB *A. baumannii* (bla OXA-24-like carbapenemase)	5	2/5
Prestel et al. (2021) – USA (FL)	Original article	07/20 -08/20	Positive	ICU/Ward	*C. auris*	35	8/35
Patel et al. (2021) – USA (MD)	Original article	05/20 -06/20	Positive	ICU/IMCU	*E. coli* ESBL CTX-M	20	Unknown
Nori et al. (2020) – USA (NYC)	Letter to the editor	03/20–04/20	Positive	ICU	*E. cloacae* Class B carbapenemase gene (blaNDM)	5	4/5
Allaw et al. (2021) – Lebanon	Original article	10/20–12/20	7/14 positive	ICU	*C. auris*	14	5/14
Shinohara et al. (2021) – Brasil	Letter to the editor	09/20–12/20	Positive	ICU	CRAB *A. baumannii*	14	7/14
Villanueva-Lozano et al. (2021) – Mexico	Letter to the editor	05/20–08/20	11/12 positive	ICU	*C. auris*	12	5/12
Garcia-Menino et al. (2020) – Spain	Original article	Not specified	Positive	ICU	*K. pneumoniae* (OXA-48, CTX-M-15)	7	1/7
Perez (2020) – USA (NJ)	Original article	02/20–07/20	17/34 positive	ICU/Ward	CRAB *A. baumanni* OXA-23 (26 isolates, 2 with Additional NDM-Metallo-betalactamase	34	10/34
Magnasco et al. (2021) – Italy	Original article	03/20–04/20	Positive	ICU	*C. auris*	6	3/6
Chowdhary et al. (2020) – India	Original article	04/20–07/20	Positive	ICU	*C. auris*	10	6/10
Duployez et al. (2021) – France	Short communication	03/20–05/20	Positive	ICU	CRAB *A. baumanni* (OXA-23)	21	5/21
De Almeida et al. (2021) -Brasil	Original article	12/20 (cross-sectional study)	8/10 positive	ICU/SICU	*C. auris*	10	3/10
Thoma et al. (2021) – Switzerland	Original article	09/20 – 11/20	7/10 positive	ICU	CRAB *A. baumanni* (OXA-23)	10	6/10

*ICU* Intensive care unit, *SICU* Semi-ICU, *NR* not reported, *Beta-L* Beta-lactam, *NDM* New Delhi metallo-beta lactamase, *VRE* Vancomycin-resistant enterococci, *CRAB* Carbapenem-resistant A. baumannii, *PPE* personal protective equipment, *AB* antibiotic

### Outbreak contributors and containment measures

**Fig. 4:**
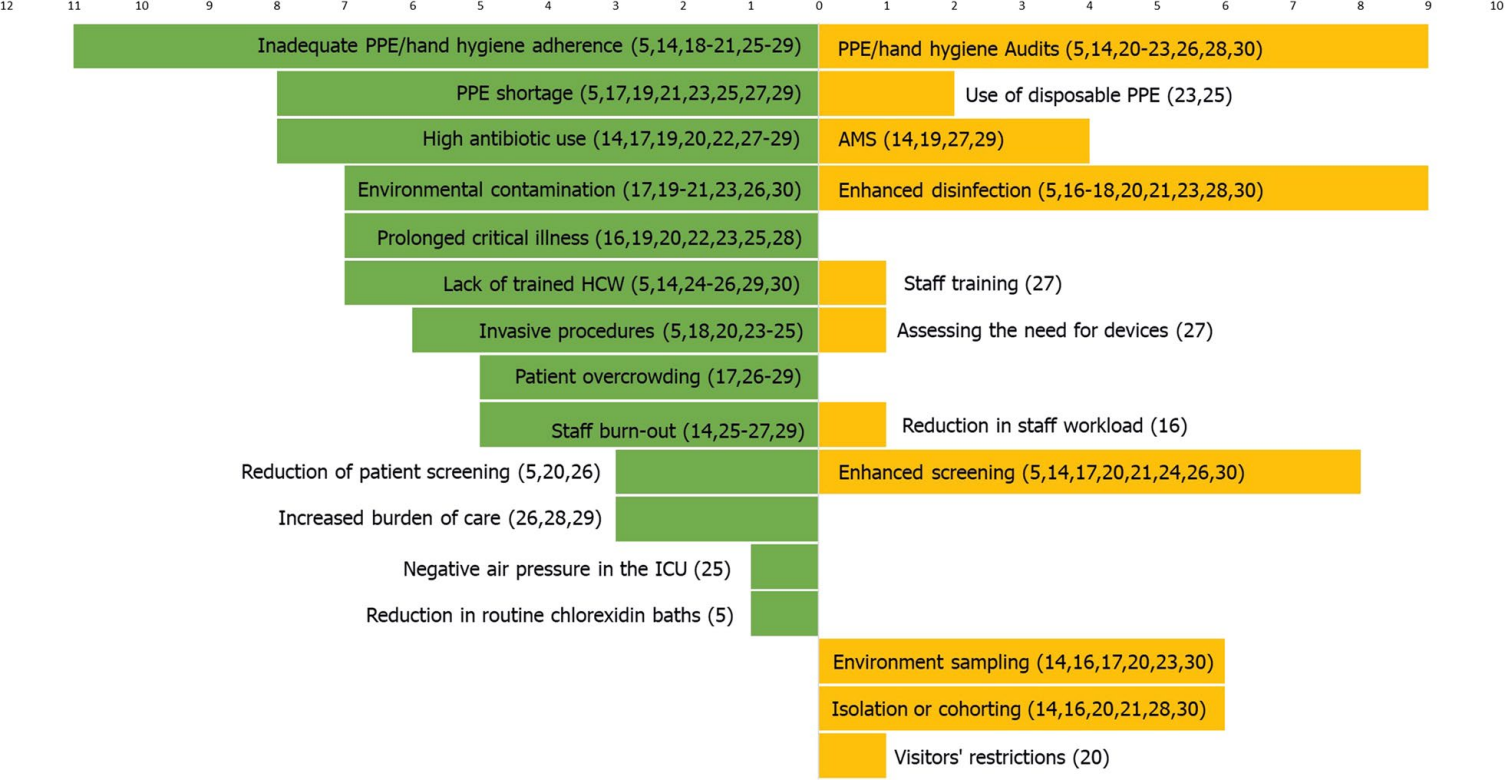
Tornado diagram showing number of outbreaks reporting specific risk factors (in green) and corresponding containment measures (in yellow). Numbers in brackets denote study references. *PPE* personal protective equipment, *HCW* health-care worker, *ICU* intensive care unit, *AMS* antimicrobial stewardship

According to the study authors, the most commonly reported risk factors potentially contributing to the outbreaks were inadequate PPE or hand hygiene adherence (reported by 11 studies), PPE shortage (8 studies) and high antibiotic use (8 studies) (Fig. 4, Additional file 1: Table S2). Other commonly mentioned risk factors included environmental contamination (7 studies) and prolonged critical illness (7 studies).

For outbreak control, studies most frequently reported enhanced environmental disinfection (n = 9) and implementation/reinforcement of PPE/hand hygiene audits (n = 9). Enhanced contact screening was reported from 8 studies. Contrasting potentially modifiable risk factors and containment measures, we identified the largest discrepancy for PPE shortage (reported by 8 studies as risk factor, addressed by 2 studies) and patient overcrowding (reported by 5 studies as risk factor, addressed by 0 studies).

### Quality of included studies

Of the 14 items of the adapted ORION statement, studies reported a median of 11 items (range 6–13) (Additional file 1: Figure S1). Four studies reported only 8 items or less.

## Discussion

We demonstrate the spread of CRAB in our ICUs during the COVID-19 pandemic within two unrelated clusters. Our systematic review shows that MDRO outbreaks have occurred worldwide during the pandemic and that *C. auris* and CRAB are most commonly implicated. We identified inadequate adherence to PPE and hand hygiene, PPE shortage, and high antibiotic use as potentially modifiable factors contributing to these outbreaks.

Our institutional CRAB outbreak is notable for two reasons. First, we observed two independent CRAB clusters, which most likely originated from patients transferred from the Balkans. Indeed, ST2-CRAB are prevalent in the Balkans, with blaOXA-23 as the predominant gene [31]. Second, except for the index patients, no other affected patient was transferred from the Balkans, making subsequent nosocomial transmission likely. While repatriations from the Balkans are common in our hospital, CRABs have only sporadically been detected in recent years. We therefore think that the particular circumstances during the COVID-19 pandemic have substantially contributed to their spread, with inadequate PPE adherence and carbapenem overuse being the most important contributing factors in our setting.

The systematic review revealed that CRAB—together with *C. auris*—are the most common pathogens causing outbreaks among COVID-19 patients. Both of these microorganisms have a high propensity to contaminate hospital environments, with the ability to survive and persist for a prolonged time on surfaces compared to other pathogens [32–34]. Indeed, environmental contamination was considered a major contributing factor in our review, and enhanced cleaning and disinfection was included in the majority of studies as containment measure. Also, *Acinetobacter sp*. and *Candida* have been identified as most common pathogens causing superinfections in COVID-19 patients according to a systematic review including 118 individual studies [35]. Here, patient factors such as prolonged length of stay, mechanical ventilation, use of broad spectrum antibiotics, and use of systemic steroids to treat COVID-19 might play a role. Of course, overrepresentation of *C. auris* and CRAB due to publication/reporting bias cannot be excluded. Also, our search strategy might have led to overrepresentation of these pathogens, although we tried to be as inclusive as possible in our literature search.

All reported outbreaks involved the intensive care setting and prolonged critical illness was mentioned in several studies as contributing factor. In line with this finding, a massive increase of Carbapenem-resistant Enterobacteriaceae (CRE) in an Italian ICU during the pandemic has been attributed to the high intensity of care for these patients; patients with prone positioning—which requires the help of four to five HCW—were significantly more likely to be colonized with CRE [6]. The critical disease state of many patients involved in these outbreaks is also reflected in the high overall mortality of 35%. However, in a meta-analysis assessing mortality of COVID-19 patients on ICUs (irrespective of bacterial superinfection), the authors found a mortality of even 41.6% [36].

According to our systematic review, inappropriate adherence to PPE and hand hygiene were the most commonly reported contributing factors. As seen in our hospital, the reinforced infection control measures put in place during the pandemic has paradoxically led in some cases to lapses in adherence to PPE and hand hygiene. Examples include failure to remove gloves after contact with patient surroundings and the use of alcohol-based sanitizer on gloved hands, which have been previously associated with increased cross-contamination [37, 38]. Also, cohorting of patients in multi-patient COVID-19 room did not always allow for the minimal safety distance between patient’s beds.

According to our review, PPE shortage was reported by several studies from across the globe. Similarly, PPE shortage has been reported by over 50% of 2′700 respondents in an international survey among intensive care personnel. The shortages in medical PPE might in some cases have led to unsafe practices such as sharing of PPE between health-care personnel, which itself increases the risk for cross-contamination [39]

A critically low HCW/patient ratio—reported as understaffing, shortage of trained HCW or patient overcrowding—was another important contributing factor in our review. A low HCW/patient ratio is a well-established risk factor for the transmission of MDRO, as shown for MRSA [40]. In addition, many hospitals had to compensate this shortage by recruiting new personnel which might have lacked the necessary competencies in infection control [6]. Of note, workload reduction—as a measure to improve the HCW/patient ratio—was only reported in one single study as part of outbreak control measures [16].

High antibiotic use in COVID-19 patients was considered a major outbreak contributor in seven studies. In fact, 75% of COVID-19 patients receive antibiotic treatment, while only 9% are actually experiencing bacterial super-infections [7]. At the same time, antibiotic stewardship (AMS) programs are currently being deprioritised in many hospitals [41], which carries the risk of increasing the selection pressure on microorganisms and facilitating the insurgence and cross-transmission of MDROs. In line with this hypothesis, the massive reduction of carbapenem consumption was one of the most important measures to control the CRAB outbreak in our hospital. Similarly, AMS was a part of successful outbreak control in three other studies.

Another particular feature of the COVID-19 pandemic was the disruption of MDRO screening surveillance programs due to constraints of personnel and financial resources. In a survey performed by the Global Antimicrobial Resistance and Use Surveillance System, 64% of the 68 participating countries reported a decrease in the number of requested screening cultures during the pandemic [42]. It remains to be seen how this influences the spread of antimicrobial resistance in the long-term.

The main limitation of our study is potential publication bias. Outbreaks with more successful outcomes and reports from settings, where involved HCW still had the resources to review and publish such outbreaks, might be overrepresented. Furthermore, some outbreak reports did not contain any information on risk factors and containment measures, which suggests reporting bias. Finally, causality cannot be inferred between reported risk factors and intervention measures. However, we still deem these aggregated experiences to be valuable for infection control specialists and also intensive care personnel for the prevention of future MDRO outbreaks during times of restricted resources.

## Conclusion

This outbreak report and systematic review show that *C. auris* and CRAB are the most frequently identified pathogens associated with MDRO outbreaks during the COVID-19 pandemic. These data also suggest that many factors which have contributed to MDRO outbreaks during the COVID-19 pandemic are potentially modifiable. These mainly include adherence to PPE and hand hygiene, PPE shortage, and antibiotic use. We as health care personnel should not let our guards down and in any case discontinue established infection prevention and control practices, as these are still the best tools at our disposal to prevent the spread of MDROs in our hospitals.

## Supplementary Information

**Additional file 1**. Supplementary material.

## Data Availability

The datasets used and/or analysed during the current study available from the corresponding author on reasonable request.

## Abbreviations

MDRO: Multidrug-resistant organism
CRAB: Carbapenem-resistant *Acinetobacter baumannii*
PPE: Personal protective equipment
HCW: Health-care worker
ICU: Intensive care unit
ESBL: Extended-spectrum beta-lactamase
CPE: Carbapenemase-producing Enterobacterales
VRE: Vancomycin-resistant Enterococci
MRSA: Methicillin-resistant *Staphylococcus aureus*
PFGE: Pulsed-field gel electrophoresis
WGS: Whole-genome sequencing
SNP: Single nucleotide polymorphism
IPC: Infection prevention and control
AMS: Antimicrobial Stewardship
