# Supplementary material for: The challenge of preventing and containing outbreaks of multidrug-resistant organisms and Candida auris during the coronavirus disease 2019 pandemic: report of a carbapenem-resistant Acinetobacter baumannii outbreak and a systematic review of the literature
Source: Antimicrob Resist Infect Control. 2022 Jan 21;11:12. doi: 10.1186/s13756-022-01052-8 (PMC8777447; doi:10.1186/s13756-022-01052-8)

**Supplementary material**

**Tabel S1:** : Search strategy. Applied terms: [“COVID-19”, “SARS-CoV-2”, “pandemic”, “Coronavirus”] AND [“Multidrug-resistant organisms”, “ESBL-E”, “CPE”, “Carbapenem-resistance”, “Acinetobacter baumannii”, “Candida auris”, “Vancomycin-resistant Enterococci”, “Methicillin-resistant Staphylococcus aureus”, “Pseudomonas aeruginosa”], and the respective acronyms.

| **Engine** | **Nb. of results** |
| --- | --- |
| MEDLINE | 399 |
| EMBASE | 606 |
| PubMed | 458 |
| Cochrane | 13 |
| CINAHL | 112 |
| NIH COVID-19 Portfolio | 418 |
| **Pool** | 2006 |
|  |  |

**Table S2**: Summary of the outbreak risk factors and control measures reported in the included studies.

| **Article** | **Outbreak risk factors** | **Other problems described** | **Control measure** | **Other measures/findings** |
| --- | --- | --- | --- | --- |
| Farfour et al. (2020) - France | Misuse of PPE; environmental contamination;  Lack of trained HCW; high burden of care for patients in prone position; absence of contact screenings; unit was used as a multiple bedroom during outbreak; overwork; | The IPC Team was unable to audit the compliance for specific IPC measures despite the misuse of gloves were reported, false security of COVID precautions. | Reinforced advice and quick training of the medical and paramedical team; contact precautions for XDR-carriers; contact screening. |  |
| Arteaga-Livias et. Al. (2021) – Peru | Decreased adherence to infection prevention and control due to overload of health system, cross contamination via the hands of the staff;  limited use of PPE (1 set of PPE to be used with all patients and throughout the shift); patient overcrowding; irrational use of antibiotics;  Staff burn out | No further audit was possible due to the work overload in all areas of our hospital | Increasing the competencies of physicians in the proper treatment of SARS-CoV-2, correct recognition of symptoms of superimposed infections; eliminating unnecessary use of antibiotics; assessing the need for the use of devices that are known to increase the probability of infection |  |
| Kampmeier et al. (2020) – Germany | Environmental contamination; understaffing. | At the time of the cluster, minimum staffing requirements in German hospitals have been suspended in order to cope with the ongoing COVID-19 | Hand hygiene training among nurses, physicians and cleaning personnel.  contact precautions including the use of PPE; intensified surface disinfection;  point prevalence screening among all patients, screening on admission and once a week for every patient; environmental sampling; patient isolation | Environmental sampling uncovered not only VRE, but also other nosocomial pathogens |
| Gottesman et al. (2021) Israel | Shortages in PPE forcing facilities to suspend the use of single-use gowns; environmental contamination; endotracheal intubation; possibly severe pneumonitis, immunosuppression due to COVID-19 and treatments | An environmental reservoir persisted in the medication room, which was not properly terminally cleaned | Hand hygiene awareness and monitoring;  environmental sampling; terminal cleaning using bleach and UVL; cohorting of CRAB patients; staff wore disposable gowns over the COVID-19 PPE overalls. | Medication room, suspected as the source of the outbreak, was closed permanently |
| Prestel et al. (2021) – USA (FL) | Inadequate use of PPE (Gowns and gloves worn during entire shift), lapses in adherence to hand hygiene; conservation strategies during anticipated or existing shortages (e.g., extended PPE wear and reuse); potentially contaminated surfaces were not always disinfected between uses. | medical supplies (e.g., oxygen tubing and gauze) were stored in open bins in hallways | Observation of health care personnel (HCP) use of personal protective equipment (PPE);  enhanced cleaning and disinfection practices; patient screening; cohorting of colonized patients | Supplies from hallways were removed |
| Patel et al. (2021) – USA (MD) | Low compliance with hand and glove hygiene and gown change; high antibiotic use;  prolonged critical illness; double occupancy of single rooms due to COVID-19 surge, tight physical spaces; team nursing model |  | Frequent infection prevention rounds to promote hand hygiene and glove and gown changes between patients;  discontinuation of double occupancy;  increased environmental services support;  attention to disinfection of reusable equipment and high-touch surfaces |  |
| Nori et al. (2020) – USA (NYC) | Challenges with adherence to infection prevention standards of care; PPE limitations; extensive antibiotic use; patient cohorting on COVID-19 units; extreme healthcare worker strain; deployment of non-traditional staff to COVID-19 units |  | Antimicrobial stewardship |  |
| Allaw et al. (2021) – Lebanon | Lack of proper hand washing; prolonged critical illness; broad spectrum antibiotics; inadequate cleaning and disinfection; catheters, mechanical ventilation; delay to report C. auris |  | Education on IPC measures, change of hand hygiene protocol, mandatory PPE use  Screening of new patients; environmental cultures; enhanced cleaning and disinfection;  visitors’ restrictions; patient isolation | Multidisciplinary meeting; terminal air decontamination |
| Shinohara et al. (2021) – Brasil | Transmission through medical equipment (ventilators, infusion pumps, and hemodialysis machines); untrained, new staff |  | Increased surveillance screening | Multidisciplinary action team |
| Villanueva-Lozano et al. (2021) – Mexico | Frequent AB and corticosteroid use in COVID-19 patients |  | Reinforcement of PPE- and  hand hygiene compliance | Multidisciplinary action team |
| Garcia-Menino et al. (2020) – Spain | PPE reuse, double gloving, disinfection of gloves;  Hiring of untrained new personnel; weakening of immune system through COVID-infection; high workload-decrease in IPC measures; more invasive procedures; negative air pressure in ICU | False security of PPE | Use of disposable gowns over PPE |  |
| Perez (2020) – USA (NJ) | suspension of gown use due to anticipated PPE Shortage, extended use of masks; decreased CRAB screening; extended use of medical equipment (ventilator circuits, suctioning catheters); reduction in routine Chlorhexidine baths; staff shortages | Suspension of routine audits for PPE use, HH & envir cleaning made identifying and correcting real-time IPC compliance issues difficult. | Enhanced PPE & HH practices with compliance audits; enhanced cleaning of surfaces with bleach; reinforcement of CRAB surveillance culturing. |  |
| Magnasco et al. (2021) – Italy | Seldom renewing in the use of PPE and sometimes shortage of the same PPE; longer median ICU Stay compared to the trimester prior the pandemic;  Clinical complexity of patients (more prone to acquiring nosocomial infections); negative impact of COVID19 on AMS programs; possible persistence of resistant pathogens in ICU. | Use of open spaces facillitates the intertransmission- | Focus on AMS and infection control |  |
| Chowdhary et al. (2020) – India | Incorrect and extended use of personal protective equipment; indwelling invasive devices such as central venous and urinary catheters |  | Enhanced cleaning of rooms with chlorine-based disinfectants at high concentrations (0.5%) |  |
| Duployez et al. (2021) – France | Increased length of ICU stay; increased duration of mechanical ventilation in COVID-19 Patients. | Search for medical device contamination was unsuccessful | Routine terminal cleaning/disinfection;  reduction in staff workload; environmental sampling; patient cohorting |  |
| De Almeida et al. (2021) -Brasil | Reuse of PPE due to shortage; high AB and antifungal use; widespread C. auris in patient surroundings; contaminated axillary thermometers; inadequate disinfection of medical equipment; patient overcrowding; high demand of mechanical ventilation; increased invasive procedures | Contaminated axillary monitoring thermometers may facilitate the dissemination of C. auris | Enhanced patient screening; environmental sampling; enhanced disinfection (Substitution of disinfection products based on quaternary ammonium by sodium hypochlorite and hydrogen peroxide‐based desinfectants) | All digital thermometers were replaced by infrared ones |
| Thoma et al. (2021) – Switzerland | Inadequate PPE adherence; excessive antibiotic prescriptions; high workload; lack of trained Staff |  | PPE/Hand hygiene Audits; patient cohorting; antibiotic stewardship; enhanced screening; environmental sampling |  |

Abbreviations: ICU. Intensive care unit; PPE, personal protective equipment; AMS, antimicrobial stewardship; CRAB, Carbapenem-resistant A. baumannii; AB, antibiotic; IPC, Infection prevention and control; HCW, Health-care worker; UVL, UV light; VRE, Vancomycin-resistant Enterococci

**Figure S1.** Risk of bias summary indicating low (+) and high (-) risk of bias according to the Orion Statement (Revman 5.4.1).
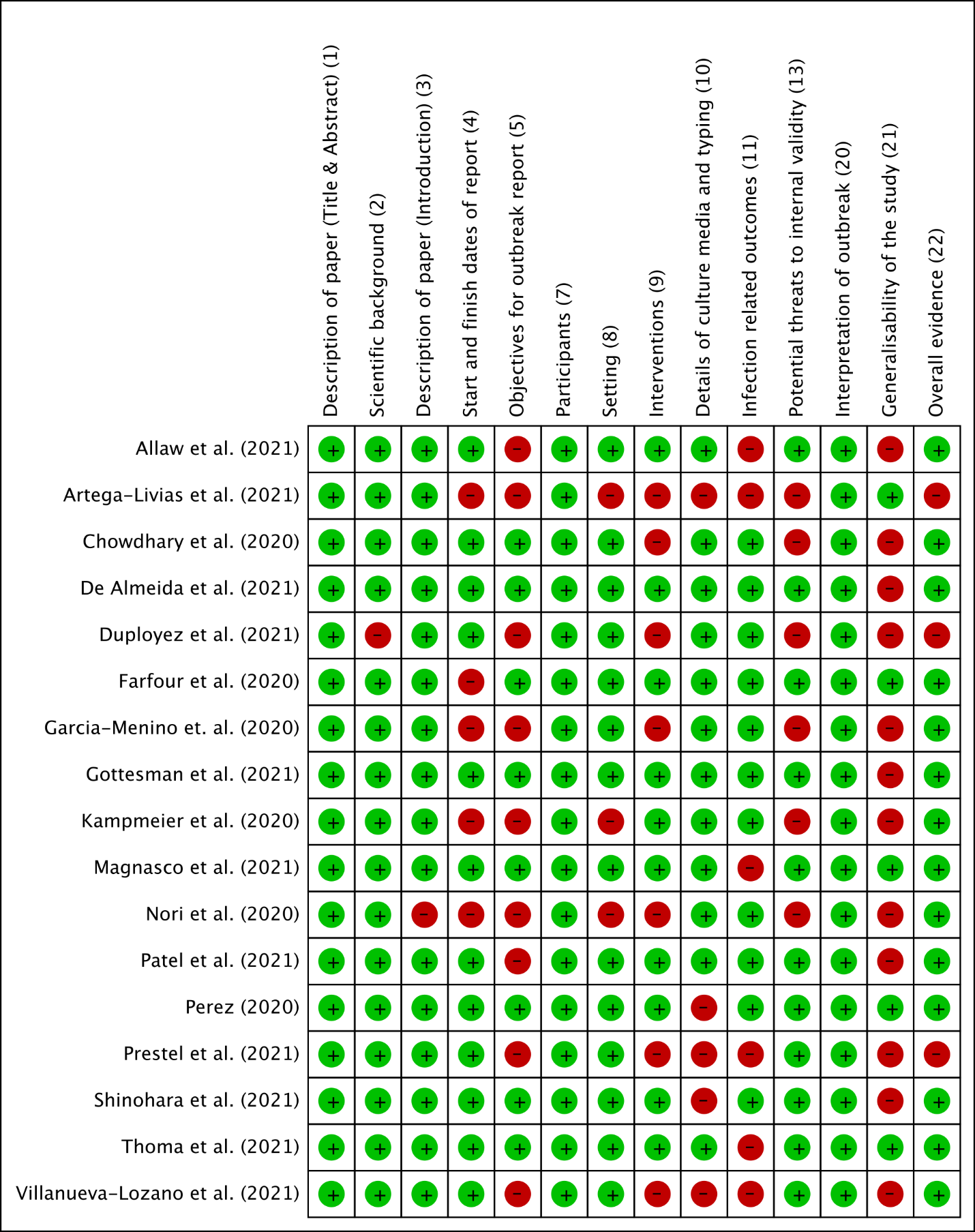

Supplement: Supplementary file 1 — Additional file 1. Supplementary material. [file 13756_2022_1052_MOESM1_ESM.docx]
